# Atteinte cérébro-méningée multiple révélant une Tuberculose multifocale chez un immunocompétent

**DOI:** 10.11604/pamj.2016.25.231.11074

**Published:** 2016-12-08

**Authors:** Tarik Boulahri, Abdellah Taous, Maha Aït Berri, Imane Traibi, Abdelhadi Rouimi

**Affiliations:** 1 Service de Neurlogie, Hopital Militaire Moulay Ismail Meknès, Meknès, Maroc

**Keywords:** Tuberculome intracrânien, méningo encéphalite, tuberculose multifocale, IRM cérébrale, Intracranial tuberculoma, meningoencephalitis, multifocal tuberculosis, brain MRI

## Abstract

La Tuberculose constitue un problème de santé publique au Maroc. L’atteinte du système nerveux central est néanmoins rare, survenant dans un contexte de tuberculose multifocale ou miliaire tuberculeuse. Cependant elle peut être un mode de révélation même chez un sujet immunocompétent. Nous rapportons le cas d’un homme de 30 ans qui avait présenté un trouble du langage évoluant dans un contexte d’altération de l’état général avec à l’examen clinique une aphasie motrice de type Broca, un syndrome pyramidal latéralisé à droite et des adénopathies latéro-cérvicales. la sérologie HIV était négative. L’IRM cérébrale: montrait des lésions associant des tuberculomes intracrâniens multiple et une image de méningo-encéphalite. La TDM thoracique montrait de multiples micronodules pulmonaires, une image cavitaire à paroi épaissie et DDB du fowler droit et du culmen. La biopsie des ganglions lymphatiques révélait la présence de granulome typique de tuberculose. Le diagnostic de tuberculose multifocale fut retenu et le patient fut mis sous traitement anti-bacillaire associé à une corticothérapie avec une bonne évolution clinico-radiologique. Cette observation est particulière par l’aspect et le siège des lésions tuberculeuse retrouvées à l’imagerie cérébrale, par l’absence d’immunodéficience, par la bonne évolution sous traitement et souligne l’intérêt de rechercher activement par un bilan exhaustif une infection tuberculeuse extra-cérébrale associée devant toute lésion cérébro-méningée évocatrice de tuberculose.

## Abstract (en)

Tuberculosis is a public health problem in Morocco. Central nervous system involvement is nevertheless rare, occurring in the context of multifocal or miliary tuberculosis. However, it may be a mode of revelation even in an immunocompetent subject. We report the case of a 30-year old man with language disorder accompanied by significant impairment of general condition. Clinical examination showed Broca’s motor aphasia, right-sided pyramidal syndrome and latero-cervical adenopathies. HIV serologic test was negative. Brain MRI showed lesions associating multiple intracranial tuberculomas and meningoencephalitis. Thoracic CT scan showed multiple pulmonary micronodules, cavity wall thickening and bronchiectasia of the right fowler and culmen. Lymph node biopsy revealed typical architecture of a TB granuloma. The diagnosis of multifocal tuberculosis was retained and the patient received anti-bacillary therapy associated with corticosteroid therapy with good clinico-radiological evolution. This study is peculiar due to the appearance and the seat of tuberculous lesions on brain imaging, the absence of immunodeficiency, a good evolution under treatment. It highlights the role of active and exhaustive assessment of associated extracerebral tuberculous infection in the case of cerebromeningeal lesion suggestive of tuberculosis.

## Introduction

La tuberculose constitue un problème majeur de santé publique dans le monde et au Maroc. L’atteinte du système nerveux central est néanmoins rare, survenant dans un contexte de tuberculose multifocale ou miliaire tuberculeuse. Cependant elle peut être un mode de révélation. Nous rapportons le cas d’une tuberculose multifocale révélée par une atteinte cérébro-méningée multiple chez un immunocompétent.

## Patient et observation

**Figure 1:**
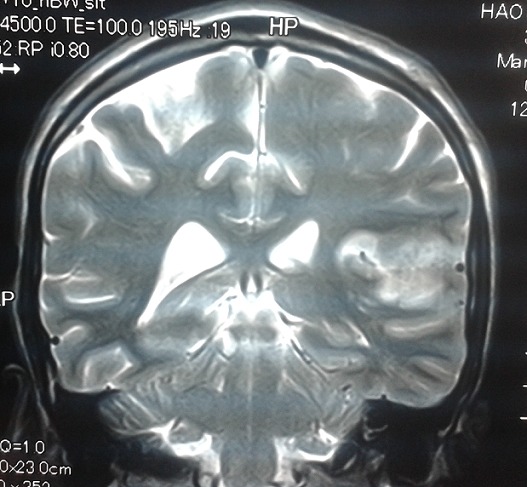
IRM cérébrale coupe coronale séquence pondéré T2 montrant: un épaississement et hypersignal T2 du cortex de l’insula et du cortex adjacent

**Figure 2:**
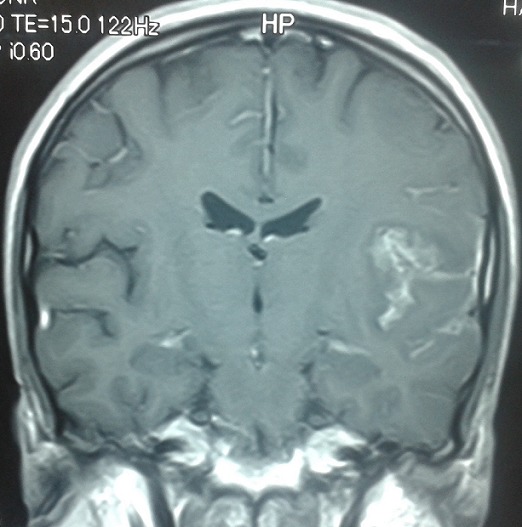
IRM cérébrale coupe coronale séquence pondéré T1 injectée montrant: prise de contraste méningée en regard du cortex de l’insula

**Figure 3:**
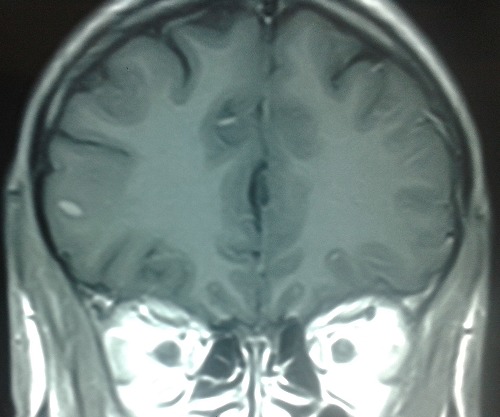
IRM cérébrale coupe coronale séquence flair montrant un tuberculome intra parenchymateux frontal gauche

**Figure 4:**
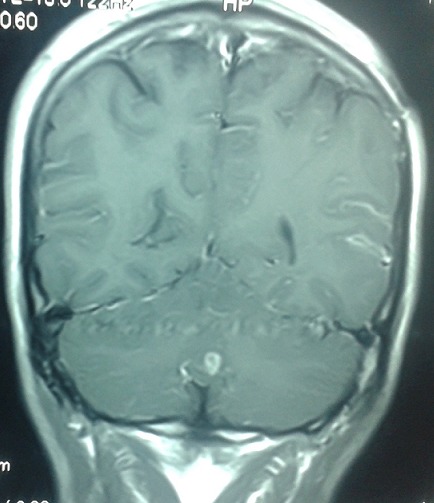
IRM cérébrale coupe coronale séquence flair montrant un tuberculome intra parenchymateux cerebelleux vermien

**Figure 5:**
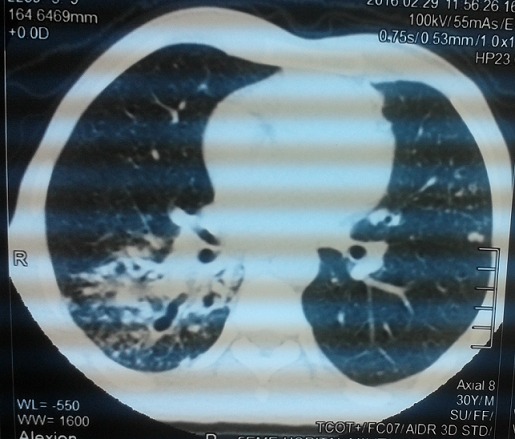
Scanner thoracique sans injection de produit de contraste, coupe axiale, fenêtre parenchymateuse montrant de multiples micronodules pulmonaires prédominant aux lobes supérieurs

**Figure 6:**
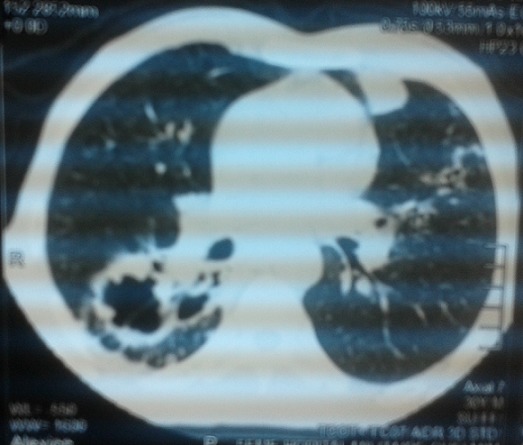
Scanner thoracique sans injection de produit de contraste, coupe axiale, fenêtre parenchymateuse montrant une image cavitaire à paroi épaissie

Un homme de 30 ans, sans antécédent de tuberculose, avait présenté 15 jours avant son admission un trouble du langage avec réduction de la fluence verbale évoluant dans un contexte d’altération de l’état général. L’examen neurologique trouvait une aphasie motrice de type Broca avec un syndrome pyramidal latéralisé à droite et des adénopathies latéro-cervicales. L’IRM cérébrale: montrait d’une part des tuberculomes intra-parenchymateux cérébraux dont un de siège frontal gauche et l’autre cérébelleux vermien d’autre part un épaississement du cortex de l’insula et du cortex adjacent avec prise de contraste intense des méninges en regard (Figure 1, Figure 2, Figure 3, Figure 4). L’étude du LCR révélait une hyperproteinorachie à 0,808 g/L, une glycorachie à 0, 305 g/L pour une glycémie de 0,957 g/L soit un rapport de 0,31% et une pléiocytose à 280 leucocytes /mm3 avec une lymphocytose à 70 %. L’examen direct et les cultures étaient négatifs. La PCR de BK dans le LCR était négative. La TDM thoracique montrait de multiples micronodules pulmonaires prédominant aux lobes supérieurs et aux segments supérieurs des lobes inferieurs, une image cavitaire à paroi épaissie et DDB du fowler droit et du culmen (Figure 5, Figure 6). La biopsie des ganglions lymphatiques révélait la présence de granulome typique de tuberculose. La recherche de BK dans les crachats était négative. La sérologie HIV était négative. L’hémogramme révélait une anémie microcytaire avec une lymphopénie à 880/UL. L’ionogramme sanguin montrait une hyponatrémie à 131 mmol/L et la CRP était à 70 mg/L. Le reste du bilan biologique était normal. Le diagnostic de tuberculose multifocale (pulmonaire, cérébrale et ganglionnaire) chez un immunocompétent fut retenu. Un traitement antituberculeux fut instauré à base d’une quadrithérapie associant isoniazide, rifampicine, pyrazinamide et éthambutol pendant 2 mois puis une bithérapie associant isoniazide et rifampicine pendant 7 mois associé à une corticothérapie adjuvante à base de prédnisolone 1mg/kg/j pendant 8 semaines avec une disparition progressive des symptômes et effacement des lésions à L’IRM cérébrale de contrôle.

## Discussion

La Tuberculose multifocale est définie par l’atteinte d’au moins deux foyers extra-pulmonaires avec ou sans atteinte pulmonaire, elle est rapportée dans 10 % des cas de tuberculose extra-pulmonaire [1] et surviennent le plus souvent chez les patients immunodéprimés, principalement porteurs du VIH [2, 3]. L’atteinte du système nerveux centrale est l’une des expressions les plus sévères de la tuberculose. Elle représente une cause majeure de morbidité et de mortalité dans les pays en voie de développement [4]. Elle est le plus souvent secondaire à une dissémination hématogène du M. tuberculosis, le plus souvent à partir d’un foyer pulmonaire (50 %), parfois à partir d’une localisation ganglionnaire, hépatique ou rachidienne [4]. Les signes cliniques de la tuberculose neuro-méningée sont polymorphe [5, 6]. La méningite tuberculeuse et le tuberculome intracrânien en sont les formes les plus fréquemment observées. Le tableau clinique complet de méningite est rarement retrouvé. La symptomatologie est celle d’une méningite fébrile associée parfois à des signes de localisation neurologique, d’installation progressive et avec des données classiques du LCR, notamment un aspect avec hypoglycorachie et hyperalbuminorachie [7, 8]. Les tuberculomes se manifestent par des atteintes focalisées d’installation progressive associées à des signes méningés [9]. La comitialité est plus rare. Une altération de la conscience peut être aussi notée, allant de l’obnubilation jusqu’au coma profond [10]. L’infection parenchymateuse dans la tuberculose est rare (6% des cas) elle peut apparaître seule ou associée à une méningite, et peut entraîner une cérébrite focale [10]. Le diagnostic de la tuberculeuse cérébro méningée repose sur des arguments biologiques, histologiques et neuroradiologiques. L’imagerie cérébrale et surtout l’IRM permet de mettre en évidence l’infarctus cérébral et son siège, l’épaississement et le rehaussement leptoméningés, la dilatation ventriculaire et fait discuter en cas de tuberculomes [11] un processus tumoral (primitif ou secondaire) ou une localisation cérébrale infectieuse (toxoplasmose, abcès bactérien ou fongique, neurocysticercose) [6, 12]. La cérébrite tuberculeuse se traduit en IRM par une zone hypointense en T1, hyperintense en T2, présentant un réhaussement gyriforme global permettant de la délimiter de l’œdème périphérique. Cette lésion se localise en périphérie et concerne le cortex et la SB. Elle se distingue d’un infarctus, éventuellement secondaire à une lésion de vascularite tuberculeuse par sa topographie ne correspondant pas à un territoire vasculaire [10]. Dans notre cas, l’IRM cérébrale montrait une atteinte cérébrale multiple associant d’ une part, des tuberculomes intra-parenchymateux cérébraux sous forme d’ anomalie de signal nodulaire frontale gauche, cérébelleuse vermienne, hypo inense T1, hyper intense T2 et Flair avec prise de contraste périphérique en anneau, et d’autre part, un aspect de méningo-encéphalite insulaire et péri insulaire gauche sous forme d’ un épaississement et hypersignal T2, flair et diffusion du cortex de l’ insula et du cortex adjacent avec prise de contraste intense des méninges en regard.

L’étude du LCR révèle la présence d’une pléiocytose à prédominance lymphocytaire, une hyperprotéinorachie et une hypoglycorachie [13] comme c’est le cas chez notre malade. Le diagnostic de certitude de la tuberculose cérébro-meningée en cas de méningite lymphocytaire repose sur la mise en évidence de bacilles tuberculeux dans le LCR sur les cultures. La PCR-BK dans le LCR est très utile pour un diagnostic rapide en attendant la culture, mais sa sensibilité n’est que de l’ordre de 32 à 45 %, avec une spécificité à 95 %. Cependant, il existe quelques faux positifs et une absence de réponse au traitement après quelques semaines doit faire reconsidérer le diagnostic [14]. Les autres examens biologiques sont non spécifiques et permettent d’éliminer les autres granulomateuses (sarcoïdose, granulomatose de Wegener, les méningites carcinomateuses). Chez ce patient, le diagnostic a été retenu devant la mise en évidence à L’examen anatomo-pathologique d’une adénopathie cervicale, d’un granulome tuberculoïde avec nécrose caséeuse. Ceci nous montre l’intérêt d’une recherche active d’une localisation tuberculeuse extra-cérébrales associée devant un contexte évocateur d’une tuberculose cérébro-méningée. Le traitement de la tuberculose multifocale est similaire à celui de la tuberculose pulmonaire; mais une durée plus prolongée, allant de 12 à 18 mois, est recommandée [15]. Le schéma thérapeutique proposé par l’OMS repose sur une quadrithérapie associant l’isoniazide, la rifampicine, la pyrazinamide et l’éthambutol (RHZE) pendant deux mois, suivis d’une bithérapie (RH) pendant sept à dix mois [13]. L’utilisation des corticoïdes est préconisée dans le traitement des tuberculomes cérébraux [15]. En effet, la corticothérapie favorise l’involution des tuberculomes et diminue le risque d’expansion paradoxale dans les premières semaines de traitement (réaction d’Hexheimer). Elle doit être administrée le plus tôt possible, dès le premier mois de traitement antituberculeux [5]. Chez ce patient, les corticoïdes ont été administrés dès le premier jour du traitement et l’évolution a été favorable.

## Conclusion

La tuberculose multifocale est une forme grave touchant habituellement les immunodéprimés. L’atteinte du système nerveux central chez un patient immunocompétent demeure rare malgré l’augmentation de l’incidence de la tuberculose. cependant elle peut être un mode de révélation d’une tuberculose multifocale comme en témoigne le cas chez notre patient d’ou l’intérêt de rechercher activement par un bilan exhaustif une infection tuberculeuse extra-cérébrale associée devant toue lésion cérébro-méningée évocatrice de tuberculose afin d’asseoir le diagnostic et débuter rapidement un traitement spécifique pour éviter les séquelles.
